# Supplementary material for: Strain-resolved de-novo metagenomic assembly of viral genomes and microbial 16S rRNAs
Source: Microbiome. 2024 Oct 1;12:187. doi: 10.1186/s40168-024-01904-y (PMC11443906; doi:10.1186/s40168-024-01904-y)
Supplement: Supplementary file 2 — Additional file 1. Supplementary Figures S1-S5 and Supplementary Note 1. [file 40168_2024_1904_MOESM1_ESM.pdf]

Supplementary Information

Strain-resolved de-novo metagenomic assembly of viral genomes and microbial 16S rRNAs

Annika Jochheim[1,2], Florian A. Jochheim[2,3], Alexandra Kolodyazhnaya[1], Étienne Morice[1,2], Martin Steinegger[4,5,6,*], and Johannes Söding[1,2,7,*]

[1]Quantitative and Computational Biology, Max-Planck Institute for Multidisciplinary Sciences, Göttingen, Germany
[2]International Max-Planck Research School for Genome Sciences, University of Göttingen, Germany.
[3]Dep. of Molecular Biology, Max-Planck Institute for Multidisciplinary Sciences, Göttingen, Germany
[4]School of Biological Sciences, Seoul National University, Seoul, South Korea
[5]Artificial Intelligence Institute, Seoul National University, Seoul, South Korea
[6]Institute of Molecular Biology and Genetics, Seoul National University, Seoul, South Korea
[7]Campus Institute Data Science (CIDAS), University of Göttingen, Germany.
[*]Correspondence: martin.steinegger@snu.ac.kr, soeding@mpinat.mpg.de

Supplementary Figures

Worst Median Best

Show heatmap

Genome statistics	penguin	megahit	metaspades	rnaspades	rnaviralspades	savage	iva	vicuna	haploflow
Genome fraction (%)	99.995	92.269	60.392	85.473	69.634	97.388	27.929	33.511	97.983
MF973193.1	99.986	96.999	88.699	99.986	96.111	99.859	83.331	99.986	99.972
MF973194.1	100	99.193	72.972	55.698	98.214	98.3	-	-	99.035
MN749156.1	100	80.832	20.008	100	15.511	94.045	-	-	94.984
Duplication ratio	1.009	1.003	1.009	1.289	1.005	1	1.055	1.005	1.01
Largest alignment	7137	6884	6295	7096	6821	7087	3527	7097	7095
Total aligned length	21 174	19 599	12 898	23 291	14 818	20 622	6227	7097	20 748
NGA50	...	...	...	...	...	...	...	...	...
LGA50	...	...	...	...	...	...	...	...	...
**Reads mapping**									
Mapped (%)	100	99.42	92.78	100	98.79	100	87.59	99.93	100
Properly paired (%)	100	98.18	89.87	100	97.62	99.31	84.69	99.93	98.52
Singletons (%)	0	0.38	1.2	0	0.46	0	1.82	0	0
Misjoint mates (%)	0	0.73	1.49	0	0.63	0.69	0.67	0	1.48
Avg. coverage depth	115	124	174	105	163	119	336	344	117
Coverage >= 1x (%)	99.15	100	100	99.8	100	100	100	100	99.52
**Misassemblies**									
# misassemblies	0	0	0	0	0	0	0	0	0
Misassembled contigs length	0	0	0	0	0	0	0	0	0
**Mismatches**									
# mismatches per 100 kbp	0	40.95	117.3	1331.57	0	0	236.73	746.9	515.71
# indels per 100 kbp	0	0	0	27.63	0	0	0	14.09	0
# N's per 100 kbp	9.36	0	0	0	0	0	0	0	0
**Statistics without reference**									
# contigs	3	5	5	7	4	3	3	1	3
Largest contig	7138	6884	6295	7127	6821	7087	3542	7128	7126
Total length	21 357	19 599	12 898	23 322	14 818	20 622	6242	7128	20 965
Total length (>= 1000 bp)	21 357	19 599	12 898	23 322	14 818	20 622	6242	7128	20 965
Total length (>= 10000 bp)	0	0	0	0	0	0	0	0	0
Total length (>= 50000 bp)	0	0	0	0	0	0	0	0	0

Figure S1: MetaQUAST assessment of assembly quality on an in-silico mixture of three HRV genomes.

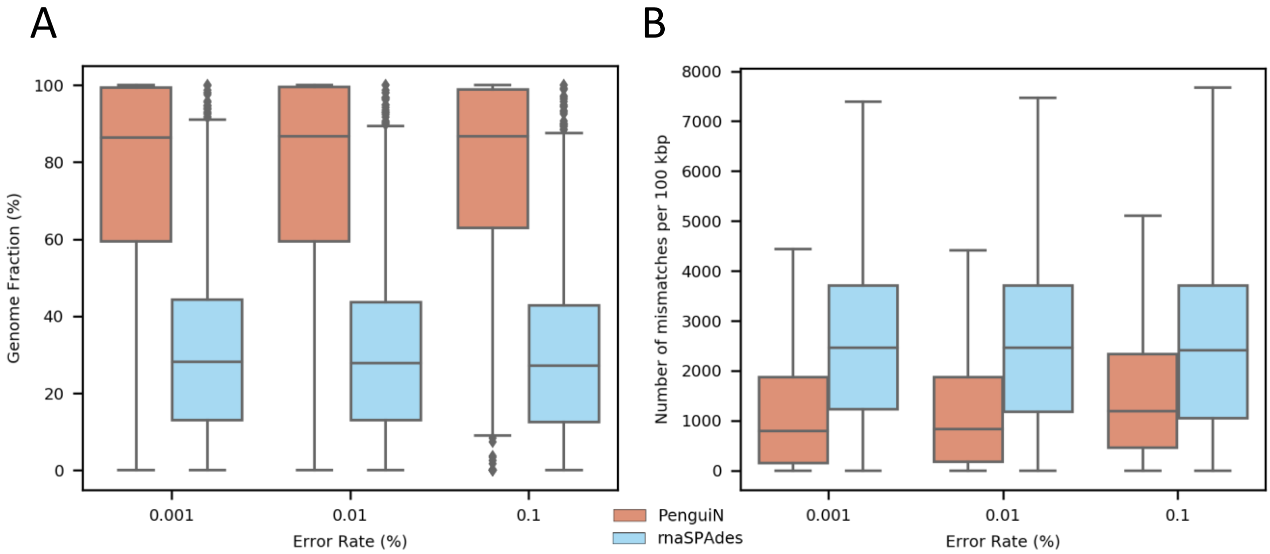

Figure S2: **Influence of sequencing errors on the assembly performance for the HIV1 10× coverage dataset evaluated by MetaQUAST.** For better visualization we did not plot the outliers in subfigure B.

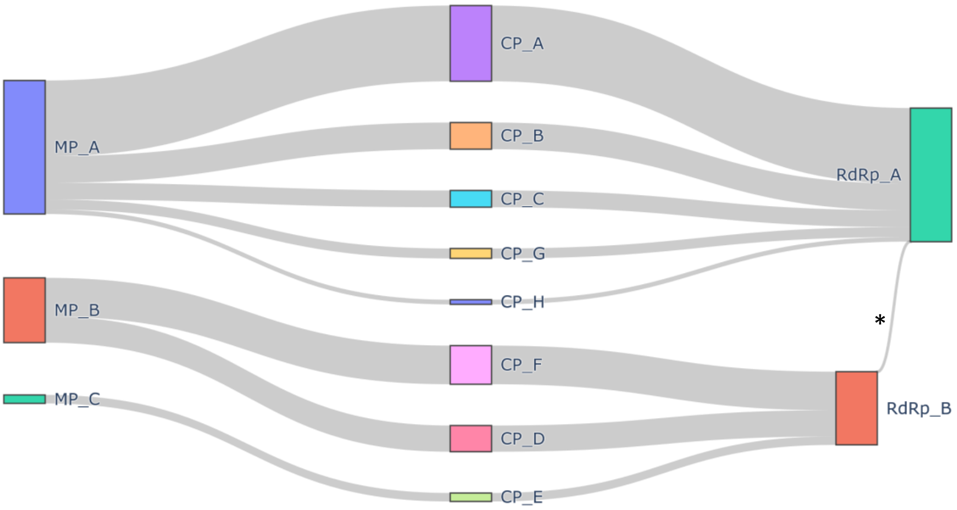

Figure S3: **Co-ooccurence profiles of the core proteins in complete ssRNA phage genomes assembled with PenguiN.** The HMM profiles released by [1] contained 3 MP, 8 CP and 2 RdRp protein clusters. Of the 48 theoretical combinations of these proteins on a complete genome, only 8 were observed in the PenguiN assembly. This is in accordance to what was seen in the Callanan et al. study. This indicates that the assembled sequences have proteins with concordant phylogenies, in contrast to what we would expect from missassemblies or chimeric sequnces. There was only one deviation marked by an asterisks (*) where a contig (contig: SRR7976310_76852) contained an additional RdRp A following the RdRp B. However, the protein classified as RdRp A (hmmscan score 469.6) in this contig was also very similar to the RdRp cluster B (hmmscan score 240.0)

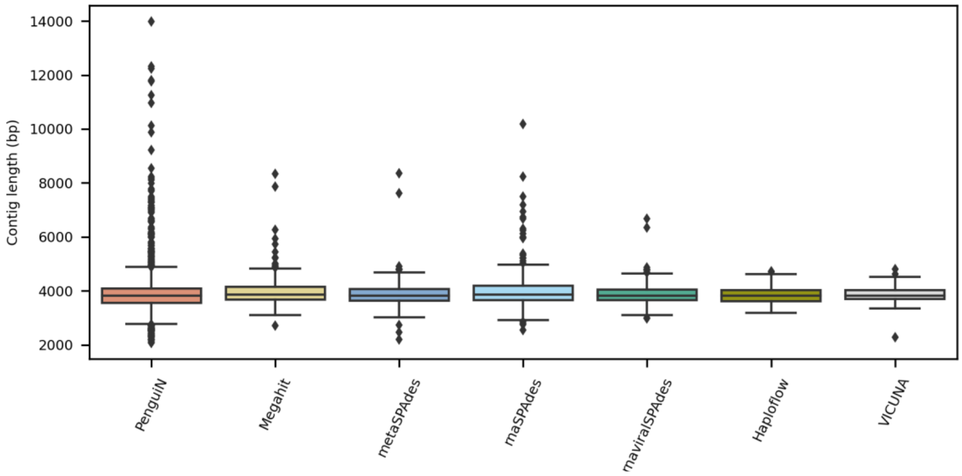

Figure S4: Length distribution of the complete ssRNA phage genomes identified from the different assemblies.

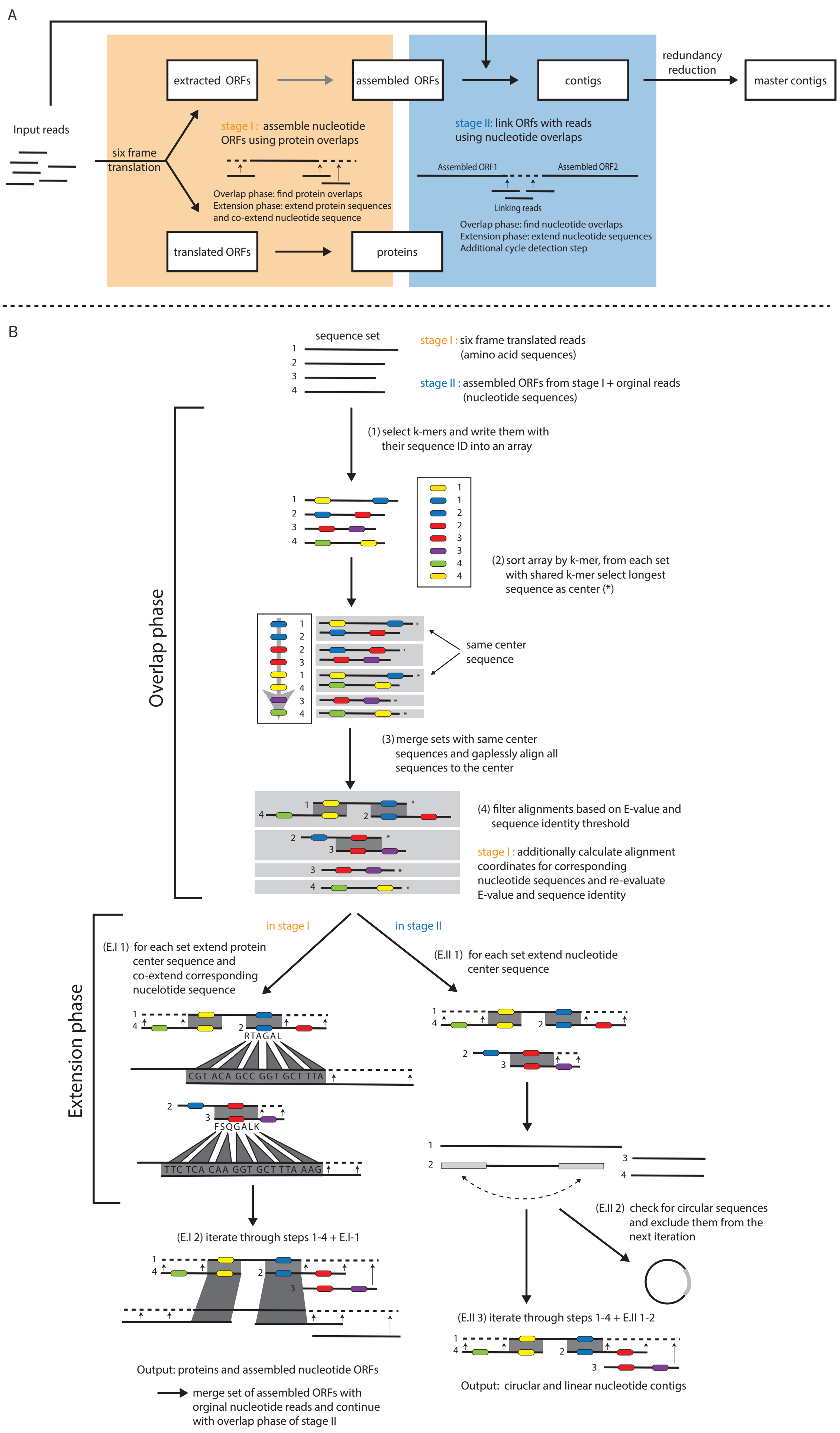

Figure S5: The stages of PenguiN's assembly in more detail

Supplementary Notes

Supplementary Note 1: Greedy extension strategy based on a Bayesian model

In the extension step, each center sequence is extended by concatenating the non-overlapping residues of the member sequences on each side ("hang-offs"), either on protein and nucleotide level simultaneously (stage 1), or on nucleotide level only (stage 2).

Even though only the member sequences with alignments to the center sequence satisfying the sequence identity threshold and the E-value criterion are considered, multiple extensions might be possible on each side of a center sequence. In the Plass [2] algorithm, the list of alignments is sorted in order of descending overlap sequence identity and the best left and right extensions are chosen. However, the length of the compared overlaps can vary greatly, which is particularly seen during the nucleotide assembly or in later iterations of the guided assembly.

For example, an overlap with length 1000 and sequence identity 98.9% might be more trustworthy than an overlap of length 100 and sequence identity 99.0% as a longer overlap also yields a statistically more significant estimation of the actual similarity between the full sequences. We used a Bayesian formulation of the problem, which can be solved analytically.

Given a query sequence (center sequence) and a target sequence (member sequence) with a mean fraction of identical residues q, and an alignment of length L between them, out of which m are matches and $L-m$ are mismatches, the probability distribution for the number of matches in the alignment is a binomial distribution

$$p(m|q,L) = \mathrm{Binom}(m|q,L) = \binom{L}{m} q^m (1-q)^{L-m} \,. \tag{1}$$

The q are hidden variables while L and m are the observed variables. Given the latter, the probability distribution of the former can be deduced using *Bayes' theorem*,

$$p(q|m,L) = \frac{p(m|q,L)\,p(q)}{\int p(m|q,L)\,p(q)\,\mathrm{d}q} \,, \tag{2}$$

where $p(q)$ is the prior probability for the fraction of matches of the alignment. We model $p(q)$ as a beta distribution,

$$p(q) = \mathrm{Beta}(q|a_q,b_q) = B(a_q,b_q)^{-1}\, q^{a_q-1}(1-q)^{b_q-1} \tag{3}$$

with

$$B(a_q,b_q) = \frac{\Gamma(a_q)\Gamma(b_q)}{\Gamma(a_q+b_q)} \,. \tag{4}$$

We therefore obtain for the hidden q

$$\begin{aligned} p(q|m,L) &\propto p(m|q,L)\,p(q) \\ &= \binom{L}{m} \frac{\Gamma(a_q+b_q)}{\Gamma(a_q)\Gamma(b_q)}\, q^{m+a_q-1}(1-q)^{L-m+b_q-1} \\ &\propto \mathrm{Beta}(q|m+a_q, L-m+b_q) \,. \end{aligned} \tag{5}$$

The proportionality constant between the left and right-hand sides must be 1 since both are normalized probability distributions.

Given two possible extensions for the same contig, described by the alignments (m_1, L_1) and (m_2, L_2), the probability that the first target sequence has lower average nucleotide identity (ANI) to the query contig than the second, is then given by

$$\begin{aligned} p(q_1 < q_2|m_1,L_1,m_2,L_2) &= \int_0^1 \int_{q_1}^1 p(q_1|m_1,L_1)\,p(q_2|m_2,L_2)\,\mathrm{d}q_2\,\mathrm{d}q_1 \\ &= \int_0^1 \int_{q_1}^1 \mathrm{Beta}(q_1|m_1+a_q, L_1-m_1+b_q)\,\mathrm{Beta}(q_2|m_2+a_q, L_2-m_2+b_q)\,\mathrm{d}q_2\,\mathrm{d}q_1 \end{aligned} \tag{6}$$

using formula (5). The probability $p(r|\mathbf{m}, \mathbf{L})$ that r is the best matching sequence extending the contig is

$$p(r|\mathbf{m}, \mathbf{L}) = \prod_{r':r' \neq r} p(q_r > q_{r'}|m_r, L_r, m_{r'}, L_{r'})\,. \tag{7}$$

To evaluate the probabilities $p(q_1 < q_2|m_1, L_1, m_2, L_2)$, we need to compute the integral

$$I := \int_0^1 \int_{q_1}^1 q_1^{\alpha-1}(1-q_1)^{\beta-1} q_2^{\alpha'-1}(1-q_2)^{\beta'-1}\,\mathrm{d}q_2\,\mathrm{d}q_1 \tag{8}$$

for

$$\alpha := m_1 + a_q, \quad \beta := L_1 - m_1 + b_q, \quad \alpha' := m_2 + a_q, \quad \beta' := L_2 - m_2 + b_q. \tag{9}$$

With a change of variables from q_2 to t, $q_2 = t + (1-t)q_1$; $t \in [0, 1]$ with $\mathrm{d}q_2 = (1-q_1)\,\mathrm{d}t$, the integral can be rewritten with fixed boundaries,

$$I = \int_0^1 \int_0^1 q_1^{\alpha-1}(1-q_1)^{\beta-1}(t+(1-t)q_1)^{\alpha'-1}\left[(1-q_1)(1-t)\right]^{\beta'-1}(1-q_1)\,\mathrm{d}t\,\mathrm{d}q_1\,. \tag{10}$$

Expanding

$$(t+(1-t)q_1)^{\alpha'-1} = \sum_{i=0}^{\alpha'-1}\binom{\alpha'-1}{i}(1-t)^i\,q_1^i\,t^{\alpha'-1-i} \tag{11}$$

yields

$$\begin{aligned} I &= \sum_{i=0}^{\alpha'-1}\binom{\alpha'-1}{i}\int_0^1\int_0^1 q_1^{\alpha+i-1}(1-q_1)^{\beta+\beta'-1}\,t^{\alpha'-i-1}(1-t)^{\beta'+i-1}\,\mathrm{d}t\,\mathrm{d}q_1 \\ &= \sum_{i=0}^{\alpha'-1}\binom{\alpha'-1}{i}\mathrm{B}(\alpha+i, \beta+\beta')\,\mathrm{B}(\alpha'-i, \beta'+i)\,. \end{aligned} \tag{12}$$

Using $n! = \Gamma(n+1)$, the probability

$$p(q_1 < q_2|m_1, L_1, m_2, L_2) = \mathrm{B}(\alpha, \beta)^{-1}\mathrm{B}(\alpha', \beta')^{-1}\,I \tag{13}$$

can then be written as

$$\begin{aligned} p(q_1 < q_2|\ldots) &= \sum_{i=0}^{\alpha'-1}\frac{\Gamma(\alpha+\beta)}{\Gamma(\alpha)\Gamma(\beta)}\frac{\Gamma(\alpha'+\beta')}{\Gamma(\alpha')\Gamma(\beta')}\frac{\Gamma(\alpha')}{\Gamma(i+1)\Gamma(\alpha'-i)}\frac{\Gamma(\alpha+i)\Gamma(\beta+\beta')}{\Gamma(\alpha+\beta+\beta'+i)}\frac{\Gamma(\alpha'-i)\Gamma(\beta'+i)}{\Gamma(\alpha'+\beta')} \\ &= \sum_{i=0}^{\alpha'-1}\frac{\Gamma(\alpha+\beta)\Gamma(\alpha+i)\Gamma(\beta+\beta')\Gamma(\beta'+i)}{\Gamma(\alpha)\Gamma(\beta)\Gamma(i+1)\Gamma(\alpha+\beta+\beta'+i)\Gamma(\beta')}\,. \end{aligned} \tag{14}$$

With a constant C and a term π_i depending on i,

$$C = \frac{\Gamma(\alpha+\beta)\Gamma(\beta+\beta')}{\Gamma(\beta)\Gamma(\alpha+\beta+\beta')} \tag{15}$$

$$\pi_i = \frac{\Gamma(\alpha+i)\Gamma(\beta'+i)\Gamma(\alpha+\beta+\beta')}{\Gamma(i+1)\Gamma(\alpha)\Gamma(\beta')\Gamma(\alpha+\beta+\beta'+i)}\,, \tag{16}$$

the probability $p(q_1 < q_2|m, L_1, m_2, L_2) = \sum_{i=0}^{\alpha'-1} C\,\pi_i$ can then be computed iteratively by

$$\pi_0 = 1 \tag{17}$$

$$\pi_i = \pi_{i-1} \times \frac{(\alpha+i-1)(\beta'+i-1)}{i(\alpha+\beta+\beta'+i-1)} = \pi_{i-1} \times r_i\,. \tag{18}$$

Based on the resulting probability value, the following decisions are made:

If $p(q_1 < q_2|m_1, L_1, m_2, L_2) > 0.55$ the (m_2, L_2) alignment is preferred, if $p(q_1 < q_2|m_1, L_1, m_2, L_2) < 0.45$ the (m_1, L_1) alignment is preferred. If the probability falls into the range 0.45 to 0.55 we consider it as "inconclusive", and prefer the extension that offers the longer extension instead.

The probability $p(q < q'|\alpha, \beta, \alpha', \beta')$ can be computed using the following pseudocode:

```
prob(a1, b1, a2, b2)
   if a1 < a2:
      return 1.0 - prob(a2, b2, a1, b1)
   sum = pi = 1.0
   for i = 1.0 to a2-1
      pi *= (a1+i-1) * (b2+i-1) / i / (a1+b1+b2+i-1)
      sum += pi
   return computeConstant(a1, b1, a2, b2) * sum
```

References

[1] J. Callanan, S. R. Stockdale, A. Shkoporov, L. A. Draper, R. P. Ross, and C. Hill, “Expansion of known ssRNA phage genomes: from tens to over a thousand,” Science Adv, vol. 6, no. 6, p. eaay5981, 2020.

[2] M. Steinegger, M. Mirdita, and J. Söding, “Protein-level assembly increases protein sequence recovery from metagenomic samples manyfold,” Nature Methods, vol. 16, no. 7, pp. 603–606, 2019.
